# The Effect of a Virtual-Reality Full-Body Illusion on Body Representation in Obesity

**DOI:** 10.3390/jcm8091330

**Published:** 2019-08-28

**Authors:** Federica Scarpina, Silvia Serino, Anouk Keizer, Alice Chirico, Massimo Scacchi, Gianluca Castelnuovo, Alessandro Mauro, Giuseppe Riva

**Affiliations:** 1 Istituto Auxologico Italiano, IRCCS, U.O. di Neurologia e Neuroriabilitazione, Ospedale S. Giuseppe, 28824 Piancavallo, Italy; 2 Istituto Auxologico Italiano, IRCCS, Laboratorio di Psicologia, Ospedale S. Giuseppe, 28824 Piancavallo, Italy; 3 Istituto Auxologico Italiano, IRCCS, Laboratorio Sperimentale di Ricerche Tecnologiche Applicate alla Psicologia, 20149 Milano, Italy; 4 Experimental Psychology/Helmholtz Institute, Faculty of Social and Behavioural Sciences, Utrecht University, 3584 Utrecht, The Netherlands; 5 Psychology Department, Università Cattolica del Sacro Cuore, 20123 Milan, Italy; 6 Istituto Auxologico Italiano, IRCCS, U.O. di Medicina Generale, Ospedale S. Giuseppe, 28824 Piancavallo, Italy; 7 Department of Clinical Sciences and Community Health, University of Milan, 20122 Milan, Italy; 8 “Rita Levi Montalcini” Department of Neuroscience, University of Turin, 10124 Turin, Italy

**Keywords:** body representation, obesity, health, virtual reality, full body illusion

## Abstract

**Background**. The effective illusory ownership over an artificial body in modulating body representations in healthy and eating disorders population has been repeatedly reported in recent literature. In this study, we extended this research in the field of obesity: specifically, we investigated whether ownership over a virtual body with a skinny abdomen might be successfully experienced by participants affected by obesity. **Methods**. Fifteen participants with obesity and fifteen healthy-weight participants took part at this study in which the VR-Full-Body Illusion was adopted. The strength of illusion was investigated through the traditional Embodiment Questionnaire, while changes in bodily experience were measured through a body size estimation task. **Results**. Participants with obesity as well as healthy-weight participants reported to experience the illusion. About the body size estimation task, both groups reported changes only in the estimation of the abdomen’s circumference after the experimental condition, in absence of any another difference. **Discussion**. Participants with obesity reported to experience the illusion over a skinny avatar, but the modulation of the bodily experience seems controversial. Future lines of research exploiting this technique for modulating body representations in obesity, specifically in terms of potential therapeutic use, were discussed.

## 1. Introduction

The efficacy of the illusory ownership over an artificial body (i.e., body-ownership illusion) as a method for modulating body representations in both healthy such as [1,2,3,4,5,6] and clinical population (such as individuals suffering of eating disorders [7,8], Complex Regional Pain Syndrome [9] and Disturbed Body Integrity [10,11] has been repeatedly reported in recent literature. Indeed, since from the traditional Rubber Hand Illusion [12], it was speculated if bodily illusions might be adopted in therapeutic settings [13,14,15,16,17] for enhancing positive rehabilitative outcomes. More recently, the research about body ownership illusions moves from the embodiment of a single body part (such as the hand [12] or the foot [10], towards the entire body. For example, in the Virtual Reality-Full Body Illusion (VR-FBI) [2,3,4,5] participants experience the illusion of ownership towards a full-body avatar. Traditionally, the avatar is showed with different physical dimensions (i.e., thinner or larger) respect to the participants. Interestingly, when participants experience successfully the illusion, they generally perceive themselves as fatter or thinner than they really are, according to the characteristics of embodied virtual body [2,3,4,5]. An example was offered by Normand and colleagues (2011) [18], who induced embodiment over a virtual body with a larger abdomen in a sample of males; immediately after this experience, participants perceived their own body as larger than the real physical dimensions, congruently with the embodied avatars’ characteristics. The illusion seemed to work also in the opposite direction: female participants who embodied avatars with skinny bodies tended to perceive their own bodies as slimmer [2,3,4,5].

As concerns eating disorders, there are in literature some interesting but preliminary studies investigating the use of body-ownership illusions as a method for exploring the boundaries of bodily self in affected individuals. For example, Eshkevari and colleagues [19] found an increased malleability of the bodily experience for individuals with eating disorders, who rated to experience the Rubber Hand Illusion higher than the healthy controls. Keizer and colleagues [7] applied the VR-FBI in a sample of individuals affected by Anorexia Nervosa, revealing that participants who experience ownership over a different virtual body reported a significant decrease in their body-size distortions too. In the 2016, our group presented a single-case study about a female individual affected by severe obesity, who experienced high level of body dissatisfaction and showed consistent distortions in the estimation of her own body-size parts [20]. We reported that both components (body dissatisfaction and body-size distortions) improved after experiencing the VR-FBI. In our knowledge, this single case study is still the only unique attempt to exploit this illusion in the context of obesity; thus, if body-ownership illusion could successfully modulate body representation in individuals affected by obesity is still an open question.

In the current study, we used a VR-FBI [1,2,20] as a technique for inducing illusory ownership over a virtual body with a skinny abdomen in a sample of individuals affected by obesity, compared to a group of healthy-weight individuals. Thus, in this study, participants saw the slim abdomen of the avatar through VR googles. When the experimenter stroked participants on their abdomen, delivering a tactile stimulation, they saw this stroking on the avatar’s abdomen (i.e., the visual input). In line with previous studies [1,2,20], two conditions were administered for all participants: the experimental condition, in which the touch and the vision of the touch were *synchronized*; the temporal coherence between the sensory input onset generally elicits the illusory ownership over the artificial body; and the control condition, in which the two perceived stimuli are *asynchronized*, since there was a temporal delay between the visual and tactile input onset. To measure the potential changes in the perceptual component of body parts representations, we adopted the Body Part Size Estimation Task [1,20]. Participants were asked to estimate their height, and the width and the circumference of three different body parts (shoulders, abdomen, and hips), at three time points: before the VR-FBI, representing the baseline, and after both the two (synchronous and asynchronous) conditions of visuo-tactile stimulation. Moreover, in line with the traditional literature [1,2,3,4,5,6,7,8,9,10,11,12,20,21], we adopted the Embodiment Questionnaire [1]: this questionnaire, administered after both stimulation conditions, allows to measure the strength of illusion in terms of: (1) *Ownership* over the virtual body; (2) *Self-location*, i.e., being in the same location of the virtual body; and (3) *Sense of agency* over the virtual body.

Considering the previous results about healthy individuals [1,2,3,4,5,18], we might expect that all participants experienced successfully the VR-FBI after the synchronous visuo-tactile stimulation (i.e., the experimental condition), but not after the asynchronous (i.e., control) condition. Moreover, about the Body Part Size Estimation Task [1,20], there would be a decrease in body size estimations compared to the baseline (i.e., before the VR-FBI took place); indeed, when the illusion emerged, healthy-weight individuals should perceive themselves slimmer than real physical dimensions, accordingly with the avatar’s dimension. Similarly, if the illusion might be efficiently induced also for individuals affected by obesity, we might expect to find a difference in the estimation of their body parts after the synchronous, but not the asynchronous condition.

## 2. Methods

The study was conducted in compliance with the Helsinki’s Declaration (of 1975, as revised in 2008), was approved by the Ethics Review Board of the Università Cattolica del Sacro Cuore (Catholic University of the Sacred Heart, Milan, Italy). Subjects volunteered to participate; they received verbal explanation of the procedures and gave informed written consent, were free to withdraw at will and were naïve to the rationale of the experiment. 

*Participants*. All participants were female and right-handed. None of them had previously experience with bodily illusions and were naïve to the rationale of this experiment. Fifteen individuals affected by obesity and fifteen female healthy-weight individuals took part in the study (Table 1). Participants affected by obesity were individuals at the first week of a diagnostic hospitalization, before a rehabilitative treatment for losing weight. We adopted the same inclusion criteria of our previous studies [22,23,24]. All subjects were nonsmokers and free from gastrointestinal, cardiovascular, psychiatric, or metabolic disorders or any concurrent medical condition not related to obesity, according to routinely clinical assessment. In addition, weight and height were measured to the nearest 0.1 kg and 0.1 cm, respectively, using standard methods. BMI was expressed as body mass (kg)/ height (m^2^). Obesity was defined for any BMI over 30 kg/m^2^. Healthy-weight participants were recruited through convenience and snowball sampling—In particular, students from the Catholic University of Milan were invited during lessons and asked to refer friends. No economical compensation was given. The same above-mentioned inclusion criteria were adopted, except for the BMI that was between 18.5 and 25 kg/m^2^.

*VR-FBI*. In this experiment we used the same protocol of our previous studies [1,2,3,4,5,6,7,8,9,10,11,12,13,14,15,16,17,18,19,20]. During the entire procedure, participants were kindly asked to avoid any movements; this set-up did not track participants’ movement and the avatar was not responsive. All participants were invited to stand upright and wear the head-mounted display (Oculus Rift DK2) to experience the VR-FBI. The Oculus Rift DK2D (compatible with the developed application) was connected to a portable computer (HP TRUE VISION with CPU Intel^®^ Core™i7). The virtual room was developed with the software Unity3D (www.unity3d.com), while the avatar was modelled using the software MakeHuman (http://www.makehuman.org/). To experience the illusion, all participants were exposed to two different conditions. During the *synchronous* condition (i.e., the experimental condition), the experimenter provided a visuo-tactile stimulation on participants’ abdomen with a brush attached to the motion-tracking device (i.e., Razer Hydra) for 90 s, while a synchronous stimulation was delivered on virtual abdomen (i.e., the virtual touch provided on the abdomen of the virtual body). In the *asynchronous* condition (i.e., the control condition) the experimenter provided the same visuo-tactile stimulation on participants’ abdomen for 90 s, but there was a delay in the corresponding virtual touch (i.e., absence of synchronization between tactile and visual stimulation). In particular, in the asynchronous condition the touch on participants’ abdomen was actually recorded by pressing a button on the Razer Hydra at the beginning of the movement. This procedure stopped the image seen by the participants as soon as the touch ended, and then it was replayed in VR when the experimenter finished each touch. Also, this stimulation lasted for 90 s.

**Figure 1:**
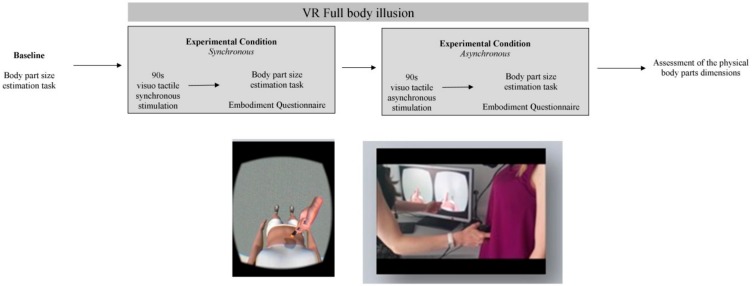
Graphical representation of the VR experimental time-line (upper part) and set-up (below up).

All participants were exposed to these two conditions in a counterbalanced order in a within subject-design. They were requested to wear the head-mounted display (i.e., Oculus) to visualize in a first-person perspective the virtual body of a female avatar by looking down at the abdomen of virtual body (i.e., the stimulated body part). They saw the body of a young woman (approximately 25 years old) with a thin abdomen (i.e., with a different shape/size in comparison to the actual body of participants) standing upright in a stimulus-free room was used to induce the full body illusion (Figure 1). 

The waist circumference of the avatar was 73.95 cm: the waist circumference of the avatar was significantly smaller respect to the mean waist circumference of both the healthy-weight participants [t(14) = 23.021; *p* < 0.001; mean = 100.18; SD = 4.41)] and participants affected by obesity [t(14) = 18.094; *p* < 0.001; mean = 140.18; SD = 14.24)]. Thus, the avatar was perceptively skinner respect to both groups of participants.

*Embodiment Questionnaire*. After both the synchronous visuo-tactile stimulation (experimental condition) and the asynchronous visuo-tactile stimulation (control condition), participants were required to fill the Embodiment Questionnaire [1], that it is routinely adopted to assess how participants subjectively experienced the illusion. It consisted of 20 items. Participants rated each statement on a 7-point Likert scale (range from 1 to 7), with higher scores indicating a stronger illusion. The questionnaire allows us to investigate the strength of illusion in terms of: (1) *ownership*, i.e., over the virtual body; (2) *self-location*, i.e., being in the same location of the virtual body; and (3) *sense of agency* over the virtual body. The three components were obtained by calculating the mean scores from the corresponding items.

*Body Part Size Estimation Task*. Before the illusion (i.e., the baseline) and after both the synchronous visuo-tactile stimulation (experimental condition) and the asynchronous visuo-tactile stimulation (control condition), participants were asked to estimate their own body parts, according to the procedure explained in our previous work [1]. Participants were asked to stand in front of a wall and to estimate the horizontal width of their own shoulders, abdomen, and hips by placing adhesive stickers: the stickers should represent the estimated distance between the left and the right side of the target body part. They were explicitly asked to not look at their body to avoid that they used it as a “reference” for making the estimates. Furthermore, they were asked to estimate the circumference of the above-mentioned three parts of their body by placing a piece of rope in a circle/oval on the floor. Moreover, participants estimated their height using an adhesive marker they placed on the wall. Also, in this case, they were explicitly asked to not use their own body as a reference to estimate their height. At the end of the entire experiment, the experimenter measured the actual width and circumference of the targeted body parts. The order of body parts (height, shoulders, abdomen, hips) and type of estimation (width, circumference) was counterbalanced over participants. For each body part and type of estimation, we calculated the percentage of misestimation according to the formula proposed by Keizer and colleagues [7]:

*percentage of misestimation* = [(estimated size − actual size)/actual size] × 100

According to the formula, a negative result suggested an underestimation, while a positive result an overestimation. 

*Data Analyses*. Differences in demographical and clinical aspects were examined through an Independent samples t-test. For the embodiment questionnaire a Repeated Measure ANOVA with the within factors of *Condition* (i.e., synchronous vs. asynchronous) and of *Subscale* (i.e., ownership, location and agency) and the between factor of *Group* (participants with obesity vs. healthy weight participants) was conducted. 

For the Body Part Size Estimation Task, we first explored any possible difference between the two groups in the estimation of the body parts at the baseline through an independent sample *t*-test. Successively, for each body part, a Repeated Measure ANOVA was run with the within factor of *Condition* (i.e., baseline, synchronous asynchronous) and the between factor of *Group* (participants with obesity vs. healthy weight participants), to verify the effect of VR_FBI on the body estimation. A significant main effect of *Condition*, and specifically between the baseline and the synchronous condition, but not between baseline and asynchronous, would suggest the effect of VR-FBI on the body size estimation; in case of significant interaction Group*Condition, the effect of *Condition* would emerge differently between individuals affected by obesity and healthy-weight individuals. In this case, for the two groups independently we performed post-hoc two independent sample t-tests: in the first one, the error in the synchronous condition was compared to the baseline; in the second, the error in the asynchronous condition. A difference was considered significant if the *p* value was below the Bonferroni’s corrected threshold of 0.025 (0.05/2). 

## 3. Results

*Demographical and clinical aspects*. Obese and healthy weight participants did not differ in *Age* and *Education level*, as showed in Table 1. As expected, participants affected by obesity had a significantly higher *BMI* than healthy weight participants. Moreover, for all considered body parts, except for height, individuals affected by obesity had higher physical dimensions than controls. 

**Figure 2:**
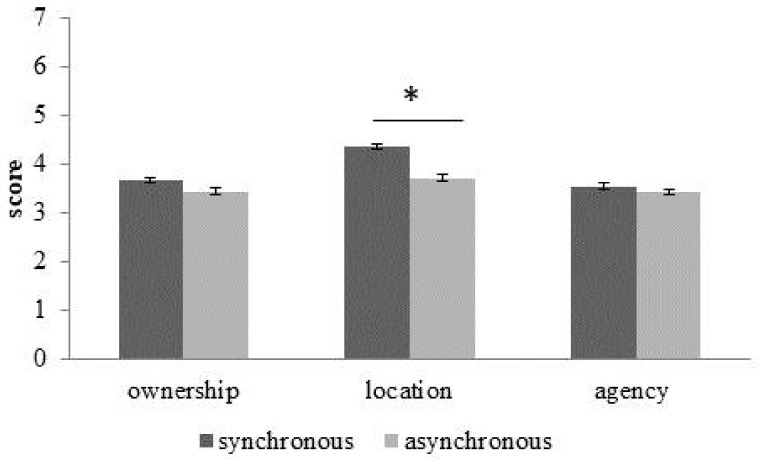
About the Embodiment questionnaire, means of the score and standard error (bars) were showed for each subscale (ownership, location and agency) in the synchronous (dark grey) and asynchronous (light grey) conditions. * indicates a significant difference according to the Bonferroni-corrected *p* value of 0.016.

*Embodiment Questionnaire*. No main effect of *Group* (participants with obesity M = 3.58; SD = 1.87; healthy-weigh participants M = 3.82; SD = 1.39) emerged [F(1,28) = 0.25; *p* = 0.62; η*ρ*^2^ = 0.009). The main effect of *Subscale* (ownership M = 3.56; SD = 1.48; location M = 4.04; SD = 1.49; agency M = 3.49; SD = 1.85) [F(2, 56) = 3.13; *p* = 0.051; η*ρ*^2^ = 0.1) was marginally significant, but not the main effect of *Condition* (synchronous M = 3.86; SD = 1.53; asynchronous M = 3.54; SD = 1.71) [F(1, 28) = 1.71; *p* = 0.2; η*ρ*^2^ = 0.05). Interestingly, an interaction Condition*Subscale [F(2, 56) = 3.8; *p* = 0.028; η*ρ*^2^ = 0.12) emerged. Thus, we performed three paired sample t-tests in which for each subscale (ownership, location and agency) we compared the scores at the two *Conditions* (synchronous vs. asynchronous). A significant difference (Bonferroni corrected p-value 0.05/3 = 0.016) emerged for the *location* subscale [t(29) = 2.69; *p* = 0.012], but not for the *ownership* subscale [t(29) = 1.32; *p* = 0.19] or for the *agency* subscale [t(29) = 0.41; *p* = 0.68] (Figure 2).

The interactions Condition*Group [F(1,28) = 0.26; *p* = 0.61; η*ρ*^2^ = 0.009) and Subscale*Group [F(2, 56) = 0.14; *p* = 0.86; η*ρ*^2^ = 0.005) were not significant. Finally the second level-interaction Group*Condition*Subscale was not significant [F(2,56) = 0.52; *p* = 0.59; η*ρ*^2^ = 0.018]. The absence of any difference between groups suggested that both participants with obesity and healthy-weight participants reported similar scores in the Embodiment Questionnaire after the induction of the VR-FBI, but it should be noticed that it is true independently from the condition (experimental/synchronous vs. control/asynchronous). However, all participants reported a stronger illusion in the experimental/synchronous condition with respect to the control/asynchronous condition in terms of self-location: in other words, when in the experimental condition, participants reported a stronger feeling to be in the same spatial location of the avatar with respect to the control condition.

*Body Parts Size Estimation Task*. As reported in the Table 1, at the baseline, analyses revealed a significant difference between the two groups in the estimation of the circumference of the shoulders, abdomen and hips, but not in the estimation of their width; moreover, no difference emerged in the estimation of the height. Specifically, as suggested by means reported in the Table 1, the healthy-weight participants showed a significant larger percentage of misestimation of the circumference of the three body parts than the participants with obesity. 

Focusing on height, a significant main effect of *Group* emerged (F(1, 26) = 4.67; *p* = 0.04; η*ρ*^2^ = 0.15), showing that individuals affected by obesity (M = 3.16; SD = 1.07) had a significant overestimation compared to the controls (M = −0.004; SD = 0.99), in absence of main effect of *Condition* (baseline M = 1.9; SD = 3.72; synchronous M = 1; SD = 4.91; asynchronous M = 1.6; SD = 4.47) (F(2, 52) = 1.82; *p* = 0.17; η*ρ*^2^ = 0.06) or a significant interaction Group*Condition (F(2,52) = 2.88; *p* = 0.88; η*ρ*^2^ = 0.41). Thus, the VR-FBI did not change the height estimation for both groups. 

**Figure 3:**
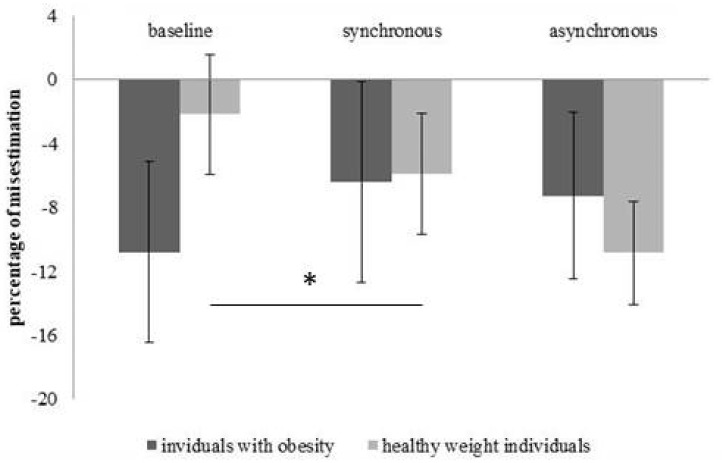
About the horizontal estimation of the shoulders, means of the percentage of misestimation and standard error (bars) were showed for participants affected by obesity (dark grey bars) and healthy-weight participants (light grey) for the three experimental conditions. * indicates a significant difference according to the Bonferroni-corrected *p* value of 0.025.

For shoulder width estimation no main effect of *Group* (participants with obesity M = −10.24; SD = 4.32; healthy-weigh participants M = −4.58; SD = 4.67) (F(1, 24) = 0.78; *p* = 0.38; η*ρ*^2^ = 0.032) or *Condition* (baseline M = −5.35; SD = 3.65; synchronous M = −8.52; SD = 3.42; asynchronous M = −8.36; SD = 3.15) (F(2,48) = 1.38; *p* = 0.26; η*ρ*^2^ = 0.055) was found. Instead, a significant interaction Group*Condition (F(2,48) = 3.5; *p* = 0.038; η*ρ*^2^ = 0.12) emerged. Post-hoc comparison for participants affected by obesity did not show a difference in the horizontal estimation of the shoulders between the baseline and the synchronous condition (t(13) = 0.049; *p* = 0.62), nor between the baseline and the asynchronous condition (t(13) = 1.13; *p* = 0.27). Instead, in the controls a significant difference emerged between the baseline and the synchronous condition (t(12) = 2.58; *p* = 0.024), but not between the baseline and the asynchronous condition (t(12) = 2.36; *p* = 0.035), when we adopted the Bonferroni corrected *p* value threshold (0.05/2 = 0.025) (Figure 3). Thus, these results show that the illusion affected shoulder width estimation of healthy-weight controls but not of individuals affected by obesity. However, as shown in Figure 3, it should be noticed that controls show a larger error after the synchronous and the asynchronous (even though the difference did not reach the significance) stimulation with respect to the baseline: this result was in contrast with the previous literature [1,2,5,18], in which it was generally reported a reduction of the error after the VR-FBI manipulation.

For width estimation of the abdomen, no main effect of *Group* (participants with obesity M = −4.49 SD = 5.39; healthy-weigh participants M = −1.29; SD = 5.79) (F(1, 26) = 0.16; *p* = 0.68; η*ρ*^2^ = 0.006) or *Condition* (baseline M = −0.006; SD = 3.77; synchronous M = −4.93; SD = 4.29; asynchronous M = −3.73; SD = 4.53) (F(2, 52) = 2.12; *p* = 0.13; η*ρ*^2^ = 0.07) emerged; moreover no significant interaction Group*Condition (F(2, 52) = 1.21; *p* = 0.3; η*ρ*^2^ = 0.04) was found. Thus, the FBI did not change the width estimation of the abdomen for both groups.

For width estimation of the hips, we found a main effect of *Group* (F(1, 24) = 8.33; *p* = 0.008; η*ρ*^2^ = 0.25): participants affected by obesity (M = 15.03; SD = 3.55) overestimated this body part with respect to the controls (M = 0.49; SD = 3.55). However, no main effect of *Condition* (baseline M = −9.47; SD = 2.91; synchronous M = 4.94; SD = 2.54; asynchronous M = 8.87; SD = 3.02) (F(2, 48) = 2.37; *p* = 0.1; η*ρ*^2^ = 0.09) or interaction Group*Condition (F(2, 48) = 2.54; *p* = 0.08; η*ρ*^2^ = 0.09) emerged. This result suggested that VR-FBI did not change the width estimation of the hips for both groups.

For circumference estimation of shoulders, a significant main effect of *Group* was found (F(1, 28) = 4.86; *p* = 0.036; η*ρ*^2^ = 0.14) confirming the previous results; in general, healthy-weight participants (M = 31.98; SD = 5.48) overestimated this body part compared to the participants affected by obesity (M = 14.88; SD = 5.48). Interestingly, a significant main effect of *Condition* emerged (F(2, 56) = 3.17; *p* = 0.049; η*ρ*^2^ = 0.10): however, when the post-hoc t-test comparisons adopting the Bonferroni corrected *p* value threshold (0.05/2 = 0.025) were performed, no significant difference between the baseline (M = 27.81; SD = 4.32) and the synchronous condition (M = 19.91; SD = 4.53) (t(29) = 2.2; *p* = 0.036) or the baseline and the asynchronous condition (M = 22.58; SD = 4) (t(29) = 1.41; *p* = 0.16) emerged. Moreover, no significant interaction Group*Condition emerged (F(1, 26) = 1.64; *p* = 0.2; η*ρ*^2^ = 0.05). 

For estimation of the circumference of the abdomen, a significant main effect of *Group* emerged (F(1, 23) = 19.95; *p* < 0.001; η*ρ*^2^ = 0.46): in line with the previous results, healthy-weight participants (M = 21.84; SD = 3) reported a significant overestimation of their body size compared to participants with obesity (M = 2.44; SD = 3.13), in absence of any main effect of *Condition* (baseline M = 13.14; SD = 2.91; synchronous M = 11.07; SD = 2.81; asynchronous M = 12.2; SD = 2.14) (F(2, 46) = 0.31; *p* = 0.73; η*ρ*^2^ = 0.013) or a significant interaction Group*Condition (F(2, 46) = 0.97; *p* = 0.38; η*ρ*^2^ = 0.04). 

**Figure 4:**
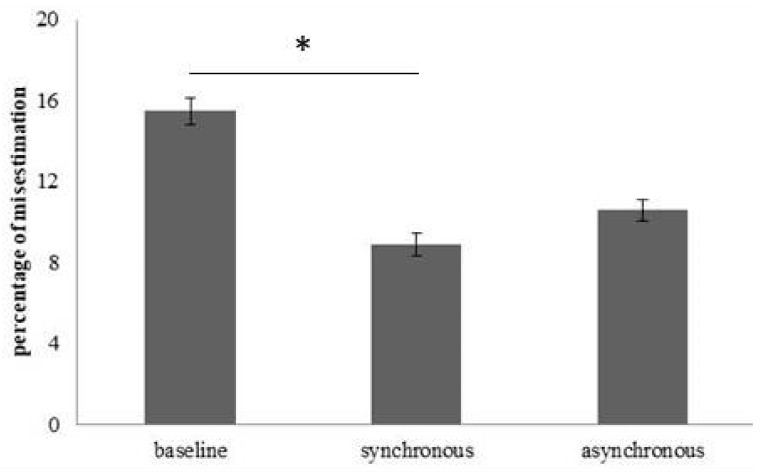
About the circumference estimation of the hips, means of the percentage of misestimation and standard error (bars) were showed for the three experimental condition (baseline, synchronous and asynchronous). * indicates a significant difference according to the Bonferroni-corrected *p* value of 0.025.

For estimation of the circumference of the hips, no main effect of *Group* (participants with obesity corrected M = 16.73; SD = 4.13; healthy-weight participants corrected M = 6.61; SD = 3.84) (F(1, 26) = 3.21; *p* = 0.08; η*ρ*^2^ = 0.11) was found. However, we observed a main effect of *Condition* (F(2, 52) = 3.51; *p* = 0.037; η*ρ*^2^ = 0.11): a significant difference between the baseline (M = 15.51; SD = 3.6) and the synchronous condition (M = 8.89; SD = 3.07) (t(28) = 2.6; *p* = 0.014) was found. Instead, no difference emerged between the baseline and the asynchronous condition (M = 10.6; SD = 2.86) (t(28) = 1.94; *p* = 0.06) (Figure 4). No significant interaction Group*Condition (F(2, 52) = 2.17; *p* = 0.12; η*ρ*^2^ = 0.07) was found. Thus, according to these results, for both groups, changes in the estimation of the hips circumference emerged after the synchronous (but not the asynchronous) condition, with respect to the baseline. Specifically, as shown in Figure 4, after the illusion was induced in the synchronous condition, all participants reported a reduction of the error in the estimation of the hips’ circumference, in line with our hypothesis.

## 4. Discussion

The aim of the present work was to investigate for the first time in literature the potential changes in body representations induced by the illusionary ownership over a virtual skinny body (i.e., the VR-FBI) in a sample of individuals affected by obesity in comparison with a group of healthy weight individuals. We adopted two measures: the Embodiment Questionnaire [1] to study the strength of the bodily illusion; and the Body Part Size Estimation Task [1] to verify if the illusion might induce a modulation of body representations in our participants.

First, we found that VR-FBI was efficiently induced in individuals with obesity with the same strength of the healthy weight individuals. Indeed, there was no significant difference between the two groups as concerns the scores obtained from the Embodiment Questionnaire [1]. However, for all participants, it should be noted that we found a significant difference in the *self-location* subscale, but not in the *ownership* and *agency* subscales, between the two experimental conditions, suggesting that also in the asynchronous condition (and thus not only the synchronous one) all individuals experience the illusion of ownership. To this regard, it was already reported that a first-person perspective of a realistic virtual body substituting participants’ own body might be sufficient to generate an illusory feeling of ownership, with changes in body representations, in absence of any multisensory stimulation [25], as reported also for the Rubber Hand Illusion [26]. Thus, the asynchronous stimulation is not a “*neutral condition*”, since it can affect body representations [27]. As concerns the self-location subscale, we did not expect specific differences between the two conditions, since our paradigm did not experimentally manipulate the spatial location. However, higher scores in self-location subscale could drive higher embodiment over the virtual body, as Maselli and Slater [25] emphasized a strict relationship between the sense of ownership and the feeling of self-location towards the virtual body. Moreover, there is an ongoing debate about what subjective components, such as body dissatisfaction [5] or bodily sensory experience [2,28,29,30]—That were not directly measured by the questionnaire—might enhance or impede to experience the bodily illusion [2,28,29,30,31]. Moreover, as recently stated by Tamè and colleagues [28] about the Rubber Hand Illusion, even though individuals report the *feeling* like the fake hand is part of their own body, “*they do not believe that it really is*”: this aspect should be taken in account also when individuals are asked to rate their experience of ownership towards an avatar.

The second other main result regarded the possible modulation of body representations, when measured through the Body Part Size Estimation Task [1], due to the bodily illusion. According to the results, an interesting pattern emerged considering the estimation of the circumference of the hips: both healthy weight individuals and individuals affected by obesity shown a reduction of the error after the synchronous, but not the asynchronous, condition, with respect to the baseline. This result seems to suggest that the illusion can efficiently modulate body representations in individuals affected by obesity, specifically in terms of the circumference of the hips, as well as in controls. In other words, affected individuals perceived themselves to have skinner hips after they have embodied a skinny avatar. However, such an effect did not emerge for the other body parts investigated in this study. How might this pattern of results be interpreted? We might observe that the hips represent a very significant and critical body part, not only in obesity but also in healthy weight participants, and specifically in women, in which there is a propensity to accumulate fat on the hips and buttocks (the “feminine gynecoid” type) instead of a more central distribution on the abdomen, that is typically observed in males [32,33,34]. Thus, it makes sense to believe that from a clinical point of view this body part is quite important and perhaps more sensitive than other body parts, such as the shoulders. Second, it should be noted that during the experimental procedure, participants looked toward the avatar’s lower part, that was clearly skinner than the own physical body dimension. Considering that, it should be carefully clarified the psychological meaning of this difference. Indeed, it cannot be excluded that the presence of a skinny avatar would might enhance (and not mitigate) the negative feelings towards the body. In this sense, the therapeutic applicability of bodily illusions in obesity should require future investigation in which also psychological and clinical factors, as well as the outcome of the weight loss program, should be taken in account. For example, it should be investigated if the alteration of body parts size estimation might be related to other psychological factors, such as fear and worries about the success of the treatment as well as the subjective level of perceived social stigma [35,36] since they could be negative consequences on efficacy of rehabilitative intervention, as well as the most recent study about the effect of bodily illusions on social cognition [31]. Indeed, Guardia and colleagues [37] described the single case of an individual affected by obesity who did not update her body image to the “new” physical body dimensions after a massive weight loss; in other words, the patient continued to describe her body as larger as before the treatment [37,38,39].

Overall, this study provided preliminary evidence that individuals with obesity can experience the VR-FBI, and that the illusion can induce a modulation of body representations only for specific body parts.

However, some caveats should be considered not only in the interpretation of our data but also for future investigation. First, a higher number of participants is mandatory to verify the reproducibility of our results.

When we investigate the application of the bodily illusion in the obesity, we should take in account not only the psychological and emotional components associated to this procedure, but also the cognitive process on which bodily illusions ground, that is the *multisensory integration* [40]. Specifically, the illusion can be efficiently induced only when the two stimuli (in this case, tactile and visual input) are perceived temporally synchronous; in other words, they are perceived as unique temporal event. For humans, multisensory integration is very critical, since it seems to be implicated in several cognitive processes, such as taste perception [41], flavor perception [42,43,44], but also bodily awareness [45,46] and sense of agency [47]. Focusing on bodily illusions, and specifically the asynchronous stimulation, the recognition of the delay between stimuli might depend on the subjective efficacy of the multisensory integration process, with effect on the induction and the strength of the illusion e.g., [25,27,48,49]. In obesity, the research about alteration of primary [50,51,52] and multiple sensory inputs [24,25,26,27,28,29,30,31,32,33,34,35,36,37,38,39,40,41,42,43,44,45,46,47,48,49,50,51,52,53,54,55,56] is still in its infancy. However, it was reported that individuals affected by obesity show an alteration of multisensory process, with possible effects of the ability in recognizing two stimuli (and overall, two events) as concurrent and concomitant [24,25,26,27,28,29,30,31,32,33,34,35,36,37,38,39,40,41,42,43,44,45,46,47,48,49,50,51,52,53,54,55,56]. Thus, future research needs to fully explore the relationship between the alteration in the experience of bodily illusions and altered multisensory integration. 

Focusing on the VR-FBI, two aspects should be taken in account. The first regards the dimension of the avatar. In this work, we adopted an avatar with a skinny body, in line with our previous studies [1,20]. However, it should be verified if different dimensions of avatar (thus, a larger avatar as well as an avatar with bodily dimensions equivalent to the physical one) would affect differently body parts size estimation. In fact, in healthy individuals, while it was reported that a correct feeling of ownership was observed for images of hands with veridical and enlarged dimension respect to the physical one in healthy individuals, but absent in case of reduced dimensions, in the Rubber Hand Illusion [57,58]. Instead, the illusion emerged with both large and small full bodies in VR-FBI [55,59]. We reported that the two groups differed in the estimation of body size at the baseline. Interestingly, the healthy weight participants reported a significant larger error for the circumference of the three body parts of shoulders, abdomen and hips in respect to the participants with obesity. As expected, for all considered body parts, except for the height, individuals affected by obesity had higher physical dimensions than controls; thus, bodily dimensions were dramatically different between groups. However, the distortion of the body parts circumference observed in the healthy weight participants was quite different respect to that of the participants with obesity. As previously mentioned, the physical reduction of body size dimensions is a goal of the rehabilitative program for losing weight, and it is reasonable to think that individuals with obesity might be more aware about their own physical body than healthy weight individuals, who otherwise are generally described to have a negative feeling and high level of concerns about their own body [60]. Moreover, it should be noted the body size distortions are highly present also in healthy population [60]. However, as reviewed by Costantini and Haggard [40], pre-existing body representations play a crucial in body ownership. Indeed, the ownership towards an external object is due not only to the simultaneity of the sensory (visual-tactile) stimulation, but also to the match between the visual image of the body part/whole body adopted in the illusion and its existing subjective cognitive representation, i.e., the perceptual body representation [61] or offline stored knowledge about own body [62]. Nevertheless, when we ask individuals to judge the dimension of body parts, perhaps we are measuring the perceptual dimension together *plus* the feelings, concerns and preoccupation about those body parts [22,23,24,25,26,27,28,29,30,31,32,33,34,35,36,37,38,39,40,41,42,43,44,45,46,47,48,49,50,51,52,53,54,55,56,57,58,59,60,61,62,63], affecting the judgement itself. The second aspect regards the fact that during this experiment, in line with previous research [2,3,4,5], participants did not move as well as avatar was static. However, brain seems to be more responsive to human action than to static images [64], with possible implications on changes in body representations [65,66]. Thus, in the future perspectives, body representations changes might be investigated when embodiment towards an avatar is induced through a visuo-motor integration (the virtual body is seen to move synchronously with the own body), instead of a visuo-tactile integration (as done in the present study), taking advantage of motion sensors technology. Nevertheless, in case of motion, the presence of cybersickness during the VR experiment should be carefully measured, specifically when clinical condition like obesity, in which dizziness and falls are generally experience [67], are tested. 

Finally, even though it is out of the scope of this work, we would like to underline some criticisms relative to the two measures (i.e., Body size estimation task and Embodiment Questionnaire) adopted in this study. About the Body parts size estimation task, it should be noticed that height (i.e., a vertical measurement taken from the top of the body to its base), horizontal (from the left of the body to the right) and circumferential (a measurement taken around the body) estimations ground on different neural mechanisms about spatial processing [68]. For example, a task in which we are required to identify the midline of a horizontal line would be solved adopting counting strategies, that instead might not be so useful when we have to estimate where is the center of a circle [69]. This aspect should be taken in account when a task about the estimation of body parts or whole body is chosen as measure of the illusion. Moreover, it should be noted that in the Body Size Estimation Task [1,2,3,4,5,6,7,8,9,10,11,12,13,14,15,16,17,18,19,20], participants are explicitly required to estimate the horizontal distance between the left and right side of each body part placing adhesive stickers on the wall. To avoid that they used their body as a reference for giving a correct body size estimation, we explicitly required participants to do not look at their own body during the task. This task has clear advantages: it’s economical and traditionally adopted in clinical settings [70,71]. However, participants are fully aware that they have to focus on their own body to solve the task, and possible overcome side effect of negative attitudes on the judgment might reduce the illusion effect. Adopting more implicit measures in which a lower level of subjective awareness about the judgment [72] is required, should strengthen our results. A similar criticism can be arisen about the Embodiment Questionnaire [1], since it allows us to assess exclusively the subjective (and thus explicit and aware) experience of the illusion. 

## 5. Conclusions

In this manuscript we presented the first investigation on the effect of a VR-FBI [2,3,4,5,17,18,20] on body representations in obesity. Indeed, only one single case study about VR-FBI on body representations in obesity [20] was reported in literature. Our results revealed that individuals affected by obesity might efficiently experience the illusory ownership over an entire virtual body, with possible changes on the estimation of the circumference of the hips. Thus, VR-FBI might be a promising tool to be adopted in rehabilitative settings [8,17], also in obesity. However, this work represents the first step in the field: future research should verify if and in which clinical and psychological circumstances as well as experimental conditions the illusion can efficiently modulate body representation in obesity.

## Figures and Tables

**Table 1:** Means and standard deviation (in brackets) were reported about demographical and clinical information, percentage of misestimation at the baseline for both groups. In bold, when *p* value was <0.05. Age and Education are reported in years; body parts physical dimensions were reported in cm.

	Participants with Obesity	Healthy Weight Participant	*t*	*p* Value	*d*
Age	32 (6)	29 (8)	0.97	0.33	0.4
Education	14 (3)	16 (1)	1.97	0.058	0.35
BMI	45 (6.69)	22 (1.66).	12.63	**<0.001**	4.71
*Body parts physical dimensions*					
Height	161.91 (8.15)	164.52 (9.31)	0.81	0.42	0.29
*Width*					
Shoulders	48.15 (7.8)	39.7 (1.82)	4.079	**<0.001**	1.49
Abdomen	42.57 (7.12)	29.7 (3.81)	6.16	**<0.001**	2.25
Hips	48.44 (6.12)	35.64 (2.6)	7.44	**<0.001**	2.72
*Circumference*					
Shoulders	121.15 (12.87)	88.7 (6.86)	8.61	**<0.001**	3.14
Abdomen	130.24 (14.73)	83.27 (9.8)	10.27	**<0.001**	3.75
Hips	140.46 (14.23)	100.17 (4.41)	10.46	**<0.001**	3.82
*Body Parts Size Estimation Task: percentage of misestimation—Baseline*					
Height	3.2 (3.73)	0.74 (4.31)	1.86	0.76	0.61
*Width*					
Shoulders	−10.8 (21.86)	−2.19 (14.54)	1.71	0.1	0.46
Abdomen	−1.6 (20.92)	0.13 (23.12)	0.22	0.82	0.07
Hips	6.8 (23.66)	10.37 (22.24)	0.62	0.5	0.15
*Circumference*					
Shoulders	16.2 (27.6)	39.45 (18.94)	2.69	**0.012**	0.98
Abdomen	6.5 (30.61)	24.43 (15.03)	3.96	**<0.001**	0.74
Hips	7.5 (22.07)	26.08 (15.84)	2.64	**0.013**	0.96
